# A combination of NMDA and AMPA receptor antagonists retards granule cell dispersion and epileptogenesis in a model of acquired epilepsy

**DOI:** 10.1038/s41598-017-12368-6

**Published:** 2017-09-22

**Authors:** Alina Schidlitzki, Friederike Twele, Rebecca Klee, Inken Waltl, Kerstin Römermann, Sonja Bröer, Sebastian Meller, Ingo Gerhauser, Vladan Rankovic, Dandan Li, Claudia Brandt, Marion Bankstahl, Kathrin Töllner, Wolfgang Löscher

**Affiliations:** 1 Department of Pharmacology, Toxicology, and Pharmacy, University of Veterinary Medicine Hannover, 30559 Hannover, Germany; 2 Center for Systems Neuroscience, 30559 Hannover, Germany; 3 Department of Pathology, University of Veterinary Medicine Hannover, Hannover, Germany; 4 Present Address: Institute for Auditory Neuroscience at University Medical Center Göttingen & German Primate Center, Göttingen, Germany

## Abstract

Epilepsy may arise following acute brain insults, but no treatments exist that prevent epilepsy in patients at risk. Here we examined whether a combination of two glutamate receptor antagonists, NBQX and ifenprodil, acting at different receptor subtypes, exerts antiepileptogenic effects in the intrahippocampal kainate mouse model of epilepsy. These drugs were administered over 5 days following kainate. Spontaneous seizures were recorded by video/EEG at different intervals up to 3 months. Initial trials showed that drug treatment during the latent period led to higher mortality than treatment after onset of epilepsy, and further, that combined therapy with both drugs caused higher mortality at doses that appear safe when used singly. We therefore refined the combined-drug protocol, using lower doses. Two weeks after kainate, significantly less mice of the NBQX/ifenprodil group exhibited electroclinical seizures compared to vehicle controls, but this effect was lost at subsequent weeks. The disease modifying effect of the treatment was associated with a transient prevention of granule cell dispersion and less neuronal degeneration in the dentate hilus. These data substantiate the involvement of altered glutamatergic transmission in the early phase of epileptogenesis. Longer treatment with NBQX and ifenprodil may shed further light on the apparent temporal relationship between dentate gyrus reorganization and development of spontaneous seizures.

## Introduction

Prevention of acquired epilepsy in patients at risk is a major unmet clinical need^1^. Some recent preclinical studies have shown that epilepsy prevention or at least disease-modification is possible in rodent models of acquired epilepsy^2,3^, but none of the reported effects has as yet been translated to patients. In view of the complexity of the processes (“epileptogenesis”) that lead to epilepsy, we have proposed that rational combinations of drugs that engage different targets presumed to be involved in the epileptogenic network, may be a more effective strategy than treatment with single, highly specific drugs^1^. Translation of such a network approach would benefit from repurposing of drugs that are clinically available.

Among the various drugs and drug targets that have been explored for antiepileptogenic effects in recent years, drugs that modulate excitatory transmission by blocking glutamate receptors of the N-methyl-D-aspartate (NMDA) subtype have been reported to exert neuroprotective effects in post-status epilepticus (post-SE) models of acquired epilepsy^2^, whereas drugs blocking the AMPA (α-amino-3-hydroxy-5-methyl-4-isoxazolepropionic acid) subtype of glutamate receptors have received relatively little attention, although AMPA receptors have long been suggested to play an important role in ictogenesis and epileptogenesis^4–7^. We reported recently that the competitive AMPA receptor antagonist NBQX (2,3-dihydroxy-6-nitro-7-sulfamoyl-benzo[f]quinoxaline-2,3-dione) did not alter development of epilepsy in the intrahippocampal kainate mouse model of epilepsy^8^, whereas an antiepileptogenic effect was observed in a rat model of neonatal seizures^9^ and in the rat amygdala kindling model of temporal lobe epilepsy (TLE)^10^.

NMDA receptors are often co-expressed in synapses with Ca^2+^-permeable AMPA receptors and co-activated simultaneously by the same neurotransmitter, L-glutamate^11^. Their close proximity in the postsynaptic density allows ionotropic and non-ionotropic crosstalk between these receptors. More than 20 years ago, we reported that the anticonvulsant effect of the AMPA receptor antagonist NBQX can be potentiated by extremely low doses (0.0001–0.1 mg/kg) of the NMDA receptor antagonist MK-801 (dizocilpine) in the amygdala kindling model of TLE^12^. Similar over-additive effects were seen when NBQX was combined with the competitive NMDA antagonist CGP39551 or the low-affinity, rapidly channel blocking NMDA receptor antagonist memantine^12,13^. Adverse effects were not potentiated by combining low doses of NMDA antagonists with NBQX. We previously also tested combinations of drugs, including ifenprodil, which act at different sites of the NMDA receptor complex, and found synergistic effects, too^14,15^.

In the present study we evaluated whether a combination of an NMDA with an AMPA receptor antagonist exerts disease-modifying or antiepileptogenic effects in the intrahippocampal kainate mouse model of mesial TLE. Recently, the first AMPA receptor antagonist, perampanel, was approved for treatment of epilepsy^6^, but we used NBQX for the present study, because our previous study on the effects of AMPA receptor antagonism on epileptogenesis was performed with NBQX^8^. As NMDA antagonist we chose ifenprodil, which inhibits NMDA receptors containing the NR2B subunit^16^. Overexpression of the NR2B subunit is thought to critically contribute to epileptogenesis in both experimental and clinical types of acquired epilepsy, both by triggering neuronal hyperexcitability and excitotoxicity and by partly mediating the proinflammatory effects of interleukin 1β (IL-1β), high-mobility group box-1 (HMGB1), and cyclooxygenase(COX)-2^17–20^. When administered alone, equivocal effects of ifenprodil have been reported for the amygdala kindling model of TLE^21,22^, and no antiepileptogenic effect was found in the pilocarpine model of TLE, although ifenprodil reduced the severity of SE-induced cell death in the hippocampus^22^. Our hypothesis was that combining ifenprodil with NBQX should block or modify epileptogenesis in the intrahippocampal kainate mouse model of mesial TLE, a widely used animal model that recapitulates many characteristics of mesial TLE in patients, including an epileptogenic focus in the hippocampus, development of spontaneous recurrent seizures (SRS), and hippocampal pathology resembling hippocampal sclerosis^23–25^.

## Materials and Methods

### Animals

Outbred male NMRI (Naval Medical Research Institute) mice, which originated from a colony of Swiss mice and are used as a general-purpose stock in many fields of research including pharmacology^26^, were obtained from Charles River (Sulzfeld, Germany) at an age of 6–7 weeks (body weight 30–40 g). Mice were adapted to the laboratory conditions for 1–2 weeks before used in experiments, so that all mice were mid-adolescent at time of kainate injection. Animals were housed under controlled conditions (ambient temperature 22–24 °C, humidity 30–50%, lights on from 6:00 am to 6:00 pm). Food (Altromin 1324 standard diet; Altromin, Lage, Germany) and water were freely available.

Experiments were performed according to the EU council directive 2010/63/EU and the German Law on Animal Protection (“Tierschutzgesetz”). Ethical approval for the study was granted by an ethical committee (according to §15 of the Tierschutzgesetz) and the governmental agency (Lower Saxony State Office for Consumer Protection and Food Safety; LAVES) responsible for approval of animal experiments in Lower Saxony (reference number for this project: 14/1659). All efforts were made to minimize both the suffering and the number of animals. A total of 76 mice were used for the present experiments.

### Intrahippocampal kainate model in mice

In this model, SE is induced by unilateral injection of kainate into the CA1 sector of the dorsal hippocampus^27,28^. For this purpose, mice were anesthetized with isoflurane (3.5% for induction, 1–2% for maintenance of anesthesia; experiment 1) or chloral hydrate (500 mg/kg i.p.; experiments 2–4) and kainate (0.21 μg in 50 nl saline; i.e., 1 nM), which was obtained from Sigma-Aldrich (Steinheim, Germany), was stereotaxically injected into the right CA1 area of the dorsal hippocampus as described previously^29^. Stereotaxic coordinates were AP, -2.1; L, −1.6; and DV, −2.3 mm from bregma, using the mouse brain atlas of Paxinos and Franklin^30^. The correct location of the injection was repeatedly approved in the different batches of NMRI mice used for the present experiments, and coordinates were adapted if needed. Kainate was slowly injected over 60 s with a 0.5 μl microsyringe. After injection of kainate, the needle of the syringe was maintained *in situ* for additional 2 min to limit reflux along the injection track. For EEG recordings, the animals were immediately implanted with bipolar electrodes aimed at the site of kainate injection in the ipsilateral CA1, using the same coordinates as for kainate injection (see Twele *et al*.^29^). During all surgical procedures and for about 1 h thereafter mice were kept on a warming pad to avoid hypothermia. Directly after surgery, mice were EEG/video monitored to verify the severity and duration of the SE induced by kainate.

In view of a relatively high loss of electrode head assemblies during the subsequent weeks after kainate in initial experiments, we improved the fixation of the head assembly by superglue (Pattex® Ultra Gel; Henkel, Düsseldorf, Germany) in subsequent experiments, which reduced the problem.

### Drug treatment

**Figure 1:**
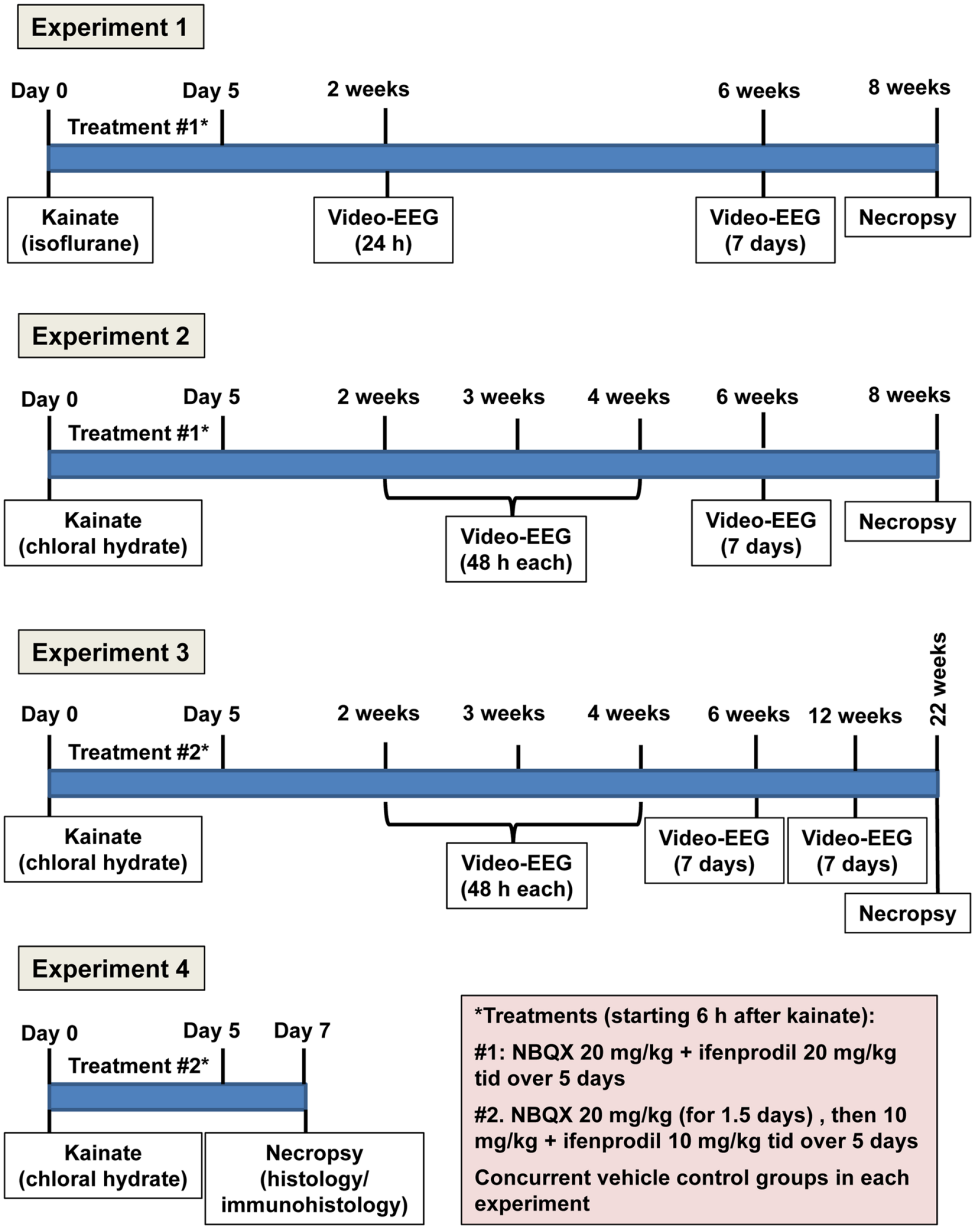
Schematic illustration of the protocol used for the four experiments performed in this study.

We have recently shown that following intrahippocampal kainate injection in male NMRI mice, there is a latent period of 5–7 days before progressive development of spontaneous electrographic and clinical seizures^29^. Therefore, mice were treated with NBQX and ifenprodil over 5 days, starting 6 h after kainate injection (Fig. 1). Before the antiepileptogenesis experiments, we evaluated the tolerability of the drug combination in a group of 6 mice that had developed epilepsy after intrahippocampal kainate. The reason for this experiment was that we had previously observed that the tolerability of NMDA and AMPA receptor antagonists in epileptic rodents is lower compared to non-epileptic rodents^8,31^. For the tolerability experiment, we chose an i.p. dose of 30 mg/kg for both NBQX and ifenprodil. The drugs were administered at 8 h intervals over 24 h, so that each mouse received 3 drug (or vehicle) injections. This dose selection was based on several previous reports. For instance, Maroso *et al*.^17^ reported that 40 mg/kg ifenprodil suppressed spontaneous seizures in the intrahippocampal kainate mouse model. Zarnowski *et al*.^32^ reported that ifenprodil increased the electroconvulsive threshold at 20 and 40 mg/kg in mice. Frasca *et al*.^18^ reported that 20 mg/kg ifenprodil were neuroprotective in a rat SE model. We recently reported that 30 mg/kg NBQX exerted an anti-seizure effect in the intrahippocampal kainate mouse model^8^. Furthermore, administration of NBQX at 20 mg/kg three times daily over 3 days, starting 6 or 8 h after intrahippocampal kainate, was tolerated without any obvious adverse effects^8^. The three times daily drug injection protocol chosen for the present experiments was based on the rapid elimination of NBQX and ifenprodil in rodents^33,34^. Furthermore, Maroso *et al*.^17^ reported that the anticonvulsant effect of ifenprodil in epileptic mice lasted for about 6 h after a dose of 40 mg/kg. For evaluating tolerability of the NBQX-ifenprodil comination, we used a strategy recently described for testing tolerability of drug cocktails in epileptic mice^35^. In short, a modified Irwin screen, the rotarod test, rectal measurement of body temperature, and measurement of body weight were repeatedly performed during the period of the experiment.

Based on the results of tolerability testing (see Results), the doses of NBQX and ifenprodil were reduced to 20 mg/kg each for the first antiepileptogenesis experiment (experiment 1; Fig. 1). In this experiment, kainate was injected under anesthesia with isoflurane, and drug (n = 8) or vehicle (n = 10) treatment was started 6 h later. Drugs were injected together (but with separate injections) three times daily over 5 days. Video-EEG monitoring was performed as shown in Fig. 1. Vehicle controls were injected three times daily with the drug vehicles (see below).

Prompted by the results of experiment 1 (see Results), in experiment 2 and all subsequent experiments, kainate was injected under anesthesia with chloral hydrate. In experiment 2, 9 mice were treated with vehicle and 10 mice with the drug combination, using the same dosing protocol as in experiment 1. Video-EEG monitoring was performed as shown in Fig. 1.

As a result of the toxicity observed in experiments 1 and 2 (see Results), the dosing protocol was changed for experiment 3 (Fig. 1). The dose of ifenprodil was reduced to 10 mg/kg three times daily. NBQX was administered at 20 mg/kg for 5 injections at 8 h intervals, then the dose was reduced to 10 mg/kg three times daily for the rest of the treatment period. Video-EEG monitoring was performed as shown in Fig. 1. Group size was 9 (drugs) and 8 (vehicle), respectively.

Experiment 4 was performed with the same dosing protocol as in experiment 3 in two groups of 8 mice for drug and vehicle treatment. In this experiment, no video/EEG monitoring was performed but mice were killed after one week following kainate for analyzing the effect of drug treatment on several brain parameters as described below. One week was chosen because inflammation is highest at 5–7 days following SE in this model (unpublished positron emission tomography data with inflammation markers) and we thought that the treatment with ifenprodil would reduce neuroinflammation as shown previously^17^.

### Video/EEG monitoring

As shown in Fig. 1, repeated periods of continuous (24 h/day) video/EEG monitoring were used to record the different types of spontaneous electrographic and clinical seizures developing after a latent period following intrahippocampal kainate in mice^29^. For EEG-recording, mice were connected via a flexible cable to a system consisting of 8 one-channel bioamplifiers (ADInstruments Ltd., Sydney, Australia) and an analog-digital converter (PowerLab 8/30 ML870 or PowerLab 4/35 PL3504/P, ADInstruments). The data were recorded (sampling rate 200 Hz, time constant 0.1 s, low pass filter of <60 Hz, 50 Hz notch filter) and analyzed with LabChart 6 or 8 for Windows software (ADInstruments). The EEG-recording was directly linked to simultaneous digital video-recording of four to eight mice per system using either one high-resolution infrared camera for up to eight mice (NYCTO Vision, CaS Business Services, Wunstorf, Germany) or four infrared board cameras (Sony) for four mice merged by one video quad processor (Monarcor TVSP-44COL). For video/EEG monitoring, mice were housed singly in clear plexiglass cages (one per cage). For monitoring during the dark phase, infrared LEDs were mounted above the cages.

As shown in Fig. 1, different intervals after kainate were chosen to compare the occurrence of spontaneous seizures in vehicle and drug treated groups. As described previously^36–38^, the highly frequent electrographic seizures occurring in this model in the ipsilateral hippocampus were differentiated into high-voltage sharp waves (HVSWs) and hippocampal paroxysmal discharges (HPDs). As recently arbitrarily defined by us for the mouse strain and model characteristics used in our studies^38^, HVSWs are characterized by sharp waves with high amplitude of at least 3-times the EEG baseline, have a duration of at least 5 s, a frequency of at least 2 Hz, and an inter-event interval of at least 3 s. During the inter-event interval, there is either no epileptic EEG activity or isolated spikes or spike trains with an amplitude of <3 times baseline occur, which was considered as interictal activity. HVSWs can either show no clear evolution or some evolution in frequency or pattern. HPDs always exhibit evolution in morphology and frequency and are often longer (>20 s) than typical HVSWs. They typically start with large amplitude HVSWs, followed by a train of lower-amplitude spikes (≥2 times baseline) of at least 5 s of increased frequency (≥5 Hz). Similar to HVSWs, during inter-event intervals (at least 3 s) no epileptic EEG activity or isolated spikes or spike trains with an amplitude of <2 times baseline are observed, which was considered as interictal activity. In addition to these typical HPDs, a second type was observed, which looked like a mixed event starting with HPD-like activity but then evolving into HVSW-like activity (see Twele *et al*.^38^) and was assigned to HPDs when counting HVSWs and HPDs. During direct observation of epileptic mice or in the videos recorded during hippocampal HVSWs and HPDs, no clear behavioral alterations were seen, but subtle alterations may have been overlooked. For comparing the frequency of HVSWs and HPDs in vehicle and drug treated mice, they were counted visually in the EEG, using four 30-min periods (at 6 a.m. and 12, 6 and 11 p.m.) during each of the recording periods shown in Fig. 1.

In addition to the highly frequent electrographic seizures, less frequent focal and generalized convulsive seizures were observed in the EEG and videos. These clinical seizures, which were all associated with paroxysmal EEG activity in the hippocampal recordings, were rated by a modified Racine scale^39^ as follows: stage 1, behavioral arrest and stereotyped sniffing; stage 2, head nodding and mouth or facial movements; stage 3, unilateral forelimb clonus; stage 4, rearing; stage 5, generalized tonic-clonic seizures with loss of righting reflexes. Stage 1–3 seizures were considered focal and stage 4 or 5 generalized convulsive seizures. For comparing the frequency of these clinical seizures in vehicle and drug treated mice, they were counted visually in the video/EEG recordings over the periods of continuous recordings shown in Fig. 1. The Racine scale was also used to rate the severity of SE induced by kainate.

All EEG and video analyses were performed in a blinded fashion, i.e., the experimenters performing the analyses were not aware whether the EEGs and corresponding videos were from drug- or vehicle-treated mice.

### Histology and immunohistochemistry

Eight weeks (experiments 1 and 2), 22 weeks (experiment 3) or one week (experiment 4) after kainate injection (see Fig. 1), the mice were anesthetized and perfused with paraformaldehyde (experiments 1 and 2), formaldehyde (experiment 3) or phosphate-buffered saline (experiment 4), depending on the subsequent immunohistological analyses. Series of coronal brain sections (40 µm in experiments 1–3, 2 µm in experiment 4) were prepared for histology and immunohistochemistry as described previously^40^. Naive age-matched groups of mice were used as controls. From experiments 2 and 3, 7 vehicle and 7 drug treated mice were randomly chosen for determining neurodegeneration in the hippocampus in thionine-stained sections at a section level of −1.94 mm from bregma. In experiment 4, neurodegeneration was determined by immunostaining with NeuN (for details see Polascheck *et al*.^41^) at three section levels (−1.34 to −1.46, −1.7 to −1.82, and −2.06 to −2.18 mm from bregma). In the thionin- and NeuN-stained sections, the left and right hippocampi were scanned in a meander-like fashion, and for detection of neurodegeneration a score system was applied to detect potential differences semi-quantitatively^42^. Scores were noted for each of the subregions of the hippocampal formation (CA1, CA2, CA3a, Ca3c, and dentate hilus): score 0, no obvious damage; score 1, abnormal appearance of the structure without clear evidence of visible neuronal loss; score 2, lesions involving 20–50% of neurons; score 3, lesions involving >50% of neurons. In addition to scoring neurodegeneration, neurons were counted in the dentate hilus of NeuN-stained sections as previously described^41^. The extent of the granule cell dispersion (GCD) was visually assessed in the thionin- and NeuN-stained sections. Visual analysis was graded with a score system: score 0 = no GCD, score 1 = mild GCD, score 2 = moderate GCD, score 3 = severe GCD.

Additional sections (at −1.7 to −1.82 mm from bregma) were stained by Fluoro-Jade C (FJC), a sensitive and specific fluorescent marker of neuronal degeneration, as described in detail previously^43^. FJC-positive neurons were counted in square fields of a defined size (90,890 μm²) that were posed subsequently in the respective region of each section at each section level to cover a large part of the region (resulting in different numbers of square fields per region): CA1, four fields; CA3a, two fields; CA3c, one field; dentate hilus, one field (for details see Polascheck *et al*.^41^).

In experiment 4, also hematoxylin + eosin (HE)-stained sections (at −1.7 to −1.82 mm from bregma) were used to measure hypercellularity in the hippocampus after kainate. The extent of hypercellularity was scored as described previously^44^: 1 = 1–25 cells, 2 = 26–50 cells, 3 =  > 50 cells per high power field.

For staining of activated microglia and brain-infiltrating macrophages, a monoclonal rat anti-Mac-3 antibody was used as described recently^40^. Mac-3 (also known as CD107b and LAMP-2) is a glycoprotein that is expressed at the plasma membrane of macrophages/microglia^45^. The amount of Mac-3-positive cells in the left and right hippocampus (at −1.58 to −1.70 mm from bregma) was assessed in a semi-quantitative fashion with the following scores: 0 = absent, no Mac-3-positive cells within the hippocampus, 1 = mild, single Mac-3-positive cells found in the hippocampus, 2 = moderate, up to 30% of hippocampal area populated with Mac-3-positive cells, 3 = severe, more than 30% of hippocampal area populated with Mac-3-positive cells.

Cells positive for interleukin (IL)-1β were stained with a rabbit polyclonal antibody (sc-7884; Santa Cruz Biotechnology, Santa Cruz, CA, USA; 1:400) using heat antigen retrieval as described in detail recently^46^. All histological and immunohistochemical analyses were performed in a blinded fashion.

### Drugs

NBQX (generously supplied by Novo Nordisk; Malov, Denmark) was freshly dissolved in distilled water (by means of dilute NaOH and gentle warming) and injected i.p. with a volume of 5 ml/kg. Ifenprodil (Tocris Bioscience, Bristol, U.K.) was dissolved in distilled water by gentle warming and injected i.p. with a volume of 5 ml/kg. Vehicle controls obtained the same volume of drug vehicles.

### Statistics

In all experiments, mice were randomly assigned to the drug and vehicle groups. Depending on whether data were normally distributed or not, either parametric or nonparametric tests were used for statistical evaluation. For comparison of two groups, either Student’s t-test or the Mann-Whitney U-test were used. In case of more than two groups we used one-way analysis of variance (ANOVA) with post hoc testing and correction for multiple comparisons. Depending on data distribution, either the ANOVA F-test, followed *post hoc* by Dunnett’s multiple comparison test, or the Kruskal-Wallis test followed *post hoc* by Dunn’s multiple comparisons test were used. Data obtained for repeated measures over time with two different groups were analyzed by two-way ANOVA with respective *post hoc* tests. For comparison of frequencies in a 2 × 2 table, Barnard’s unconditional test^47^ was used, because this test preserves the significance level and generally is more powerful than Fisher’s exact test for moderate to small samples^48^. Except Barnard’s unconditional test, all statistical analyses were performed with the Prism 6 software from GraphPad (La Jolla, CA, USA). Two-sided tests were used; a P ≤ 0.05 was considered significant.

## Results

### Tolerability testing before the antiepileptogenesis experiments

In order to reduce the risk of severe adverse effects during the antiepileptogenesis experiments, tolerability of a combination of NBQX (30 mg/kg) and ifenprodil (30 mg/kg) was tested in 6 mice that had developed epilepsy after intrahippocampal kainate. The drugs were administered i.p. at 8 h intervals over 24 h, so that each mouse received 3 drug injections. Vehicle-treated mice were used as control. As previously described^35^, a battery of observational tests was repeatedly used to evaluate tolerability. The predominant adverse effects were sedation and moderate to severe ataxia that were maximal 30 min after injection and rapidly disappeared thereafter. Furthermore, a decrease in body temperature was observed one h after drug administration. Based on the adverse effects observed in this experiment, it was decided to decrease the doses of both compounds to 20 mg/kg for the antiepileptogenesis experiments. In contrast to our expectations, severe problems with tolerability occurred in these subsequent experiments.

### Tolerability in antiepileptogenesis experiment 1

As shown in Fig. 1, kainate was injected under anesthesia with isoflurane in 18 mice; 6 h later, treatment with vehicle or NBQX + ifenprodil (20 + 20 mg/kg) was started in 10 and 8 mice, respectively. As reported previously^29^, the kainate-induced SE, which developed when the inhalative administration of isoflurane was stopped with a latency of about 2.5 h after kainate injection, was very severe with frequently occurring generalized convulsive tonic-clonic seizures and running and bouncing in addition to the typical limbic seizures induced by intrahippocampal kainate. During treatment with NBQX + ifenprodil (three times daily over 5 days), 3/8 mice died (one each on day 3, 5, and 9 post-SE) compared to 1/10 mice of the vehicle control group (P = 0.1139). Respiratory problems were observed in the drug treated mice. In addition to the loss of mice by mortality, several mice lost their electrode head assembly during the subsequent weeks of the experiment, so that the group size became too small for any meaningful group comparison of seizure frequencies.

We concluded that the severe SE plus the adverse effects of the treatment led to enhanced mortality, because no such mortality had been observed in the preliminary tolerability experiments with higher doses of both compounds. Thus, based on recent experiments using different anesthetic drugs during intrahippocampal kainate injection^29^, we decided to repeat the experiment with chloral hydrate.

### Tolerability in antiepileptogenesis experiment 2

As shown in Fig. 1, in this experiment kainate was injected under anesthesia with chloral hydrate in 19 mice; 6 h later, treatment with vehicle or NBQX + ifenprodil (20 + 20 mg/kg) was started in 9 and 10 mice, respectively. Because of the long-lasting effect of this anesthetic drug, mice were heavily sedated over hours, and a long interval (~5 h) between kainate injection and SE onset was observed. Most likely because of the long-lasting sedative effect of chloral hydrate, SE severity was lower compared to anesthesia with isoflurane. Despite the lower SE severity, 6/10 drug treated mice died during treatment (one mouse on day 2, three on day 3, one on day 4, and one on day 5 post-SE) vs. 1/9 vehicle treated mice (on day 5 post-SE), resulting in a significant difference in mortality between groups (P = 0.0163). If mortalities from experiments 1 and 2 were combined, 2/19 vehicle treated mice died vs. 9/18 drug treated mice, which was a highly significant difference (P = 0.0110).

As in experiment 1, respiratory problems were observed during drug treatment in experiment 2. Furthermore, several mice of both groups lost their head assemblies during subsequent weeks, so that only 3 vehicle-treated and 4 drug-treated mice could be video/EEG recorded over 6 weeks as shown in Fig. 1. Based on these data, we decided to change the dosing schedule of the drug treatment and performed a third experiment.

### Tolerability in antiepileptogenesis experiment 3

In this experiment, in which kainate was injected under anesthesia with chloral hydrate in 17 mice, the dose of ifenprodil was reduced to 10 mg/kg. NBQX was administered at 20 mg/kg for 1.5 days, then the dose was reduced to 10 mg/kg for the rest of the treatment period (Fig. 1). In contrast to experiments 1 and 2, none of the drug-treated mice (n = 9) died during treatment, and the respiratory problems observed with the higher doses were not observed with this treatment protocol. We also managed to reduce the number of head assembly losses (see Methods), so that 6 vehicle-treated and 5 drug-treated mice could be video-EEG monitored over 6 weeks.

### The combination of NBQX and ifenprodil retards epileptogenesis

To increase the power of the study, we combined mice from experiment 2 and 3 for final analysis of video/EEG data. Statistical comparison of seizure incidence or frequencies from the two groups did not indicate any significant difference between experiments. In both groups, fewer of the treated mice exhibited clinical seizures than controls at 2 weeks after kainate (median seizure frequency was decreased from 1/48 h in vehicle controls to 0/48 h in drug treated mice in both experiments). However, one problem with experiment 2 may be that those animals given treatment that died were those with worse SE, resulting in an inherent selection bias in the drug treated group towards a less severe insult. We therefore analyzed whether mice that died in experiment 2 had a longer or more severe SE than animals that did not die. For this purpose, the video/EEG recorded during SE was analyzed. Mean duration of SE was 21.1 h (range 18.4–22.5 h) in the animals of experiment 2 without significant difference between mice that survived (19.8) or died (21.7). As reported previously^8,28,29,36^, in all mice the limbic SE was characterized by paroxysmal activity (continuous activity of spikes or spike and waves and polyspikes) in the hippocampal EEG, which was irregularly interrupted by electroclinical focal or generalized convulsive seizures. The mean number of focal stage I-III and secondarily generalized convulsive stage IV/ V seizures during SE was 3.5 (range 0–9) and 1.25 (range 0–5) in the whole group, again without significant differences between mice that survived or died after SE.

**Figure 2:**
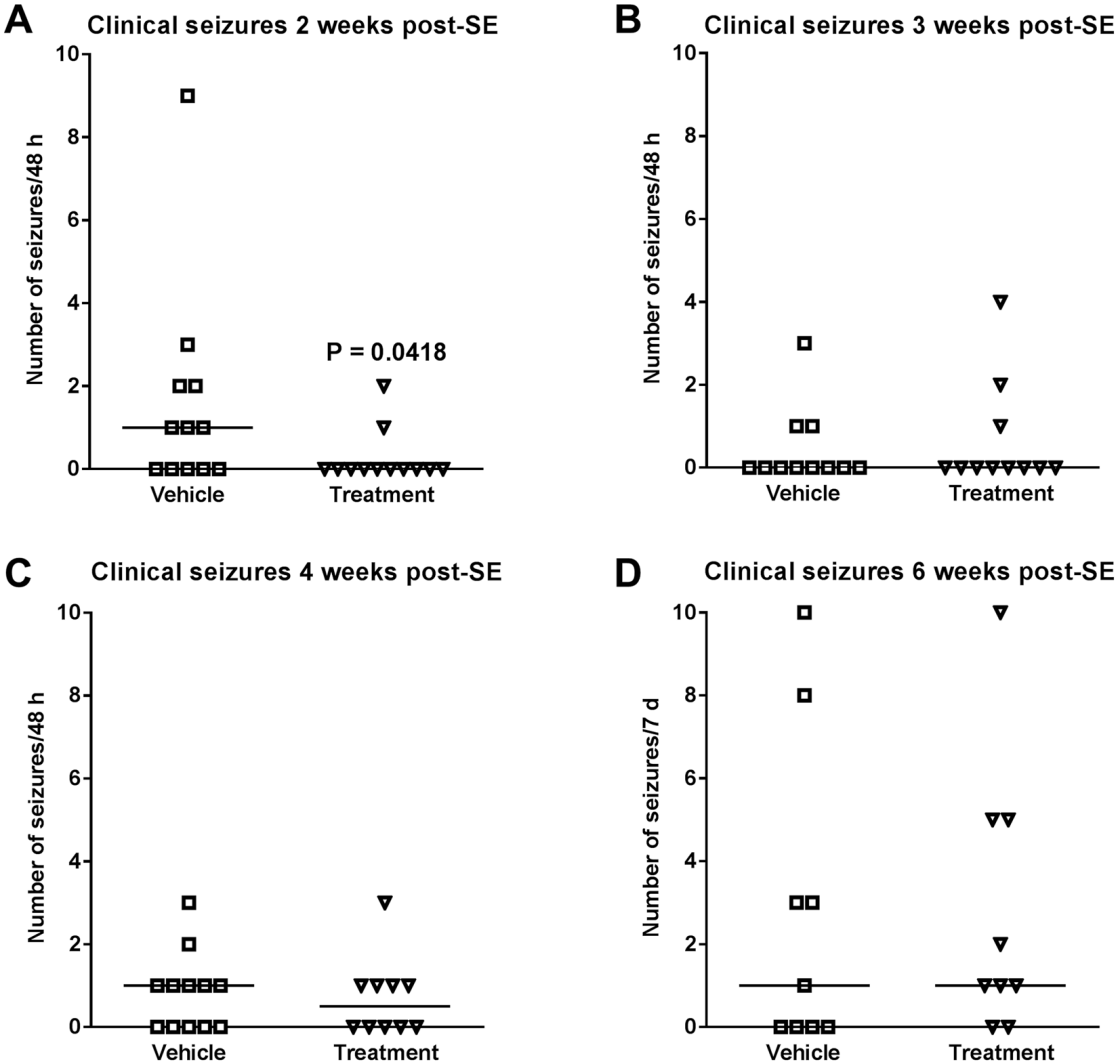
Treatment with a combination of NBQX and ifenprodil during the latent period of the intrahippocampal kainate mouse model retards the development of spontaneous clinical seizures. Data are combined from experiments 2 and 3 (see Fig. 1) and are shown for 4 periods (2, 3, 4, 6 weeks) after kainate. In each graph, the number of clinical seizures in individual mice is shown; the group median is indicated by the horizontal line. Analysis of data by two-way ANOVA indicated a significant difference between vehicle and drug treated groups (P < 0.05); *post hoc* analysis by Dunn’s multiple comparisons test indicated that drug treatment significantly reduced seizure frequency at 2 weeks after kainate (P = 0.0418).

**Figure 3:**
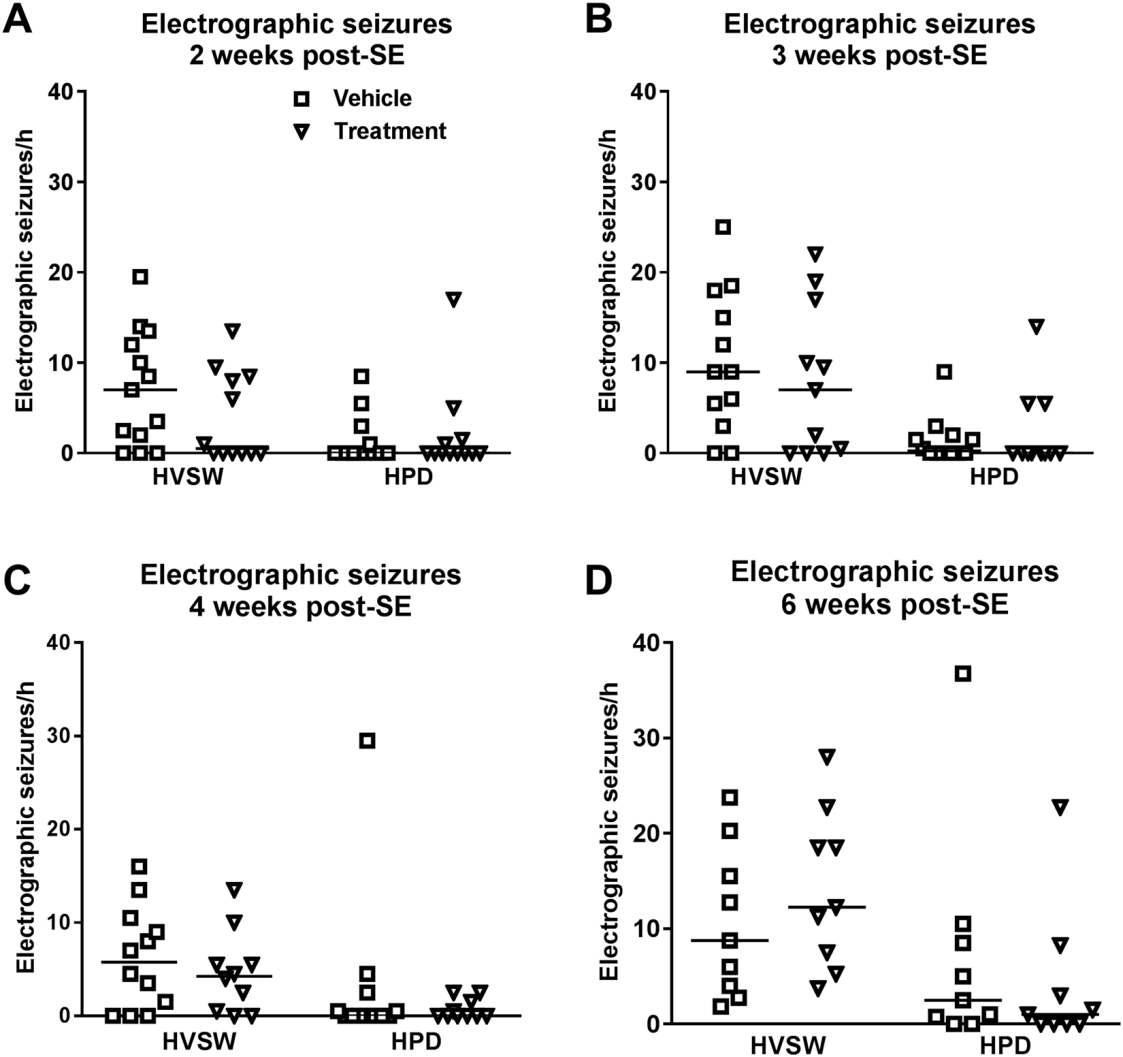
Treatment with a combination of NBQX and ifenprodil during the latent period of the intrahippocampal kainate mouse model does not retard the development of spontaneous electrographic seizures. Data are combined from experiments 2 and 3 (see Fig. 1) and are shown for 4 periods (2, 3, 4, 6 weeks) after kainate. In each graph, the number of electrographic seizures in individual mice is shown; the group median is indicated by the horizontal line. Two types of electrographic seizures are shown: high-voltage sharp waves (HVSWs) and hippocampal paroxysmal discharges (HPDs).

When data of experiments 2 and 3 were combined for analysis of potential antiepileptogenic or disease-modifying effects of drug treatment, the drug combination significantly reduced the frequency of clinical seizures at 2 weeks after kainate (Fig. 2A). Furthermore, the number of mice exhibiting such seizures was reduced by drug treatment: whereas 7/12 vehicle-treated mice exhibited clinical seizures, this was observed in only 2/12 drug treated mice (P = 0.0430) at two weeks after kainate. Most of the mice of both groups exhibited highly frequent electrographic seizures, although a tendency for reduced frequency of such seizures (HVSWs) was observed in drug-treated mice at two weeks after kainate (Fig. 3A). Ten of 13 vehicle controls exhibited HVSWs compared to 6/12 drug-treated mice (P = 0.0913).

**Figure 4:**
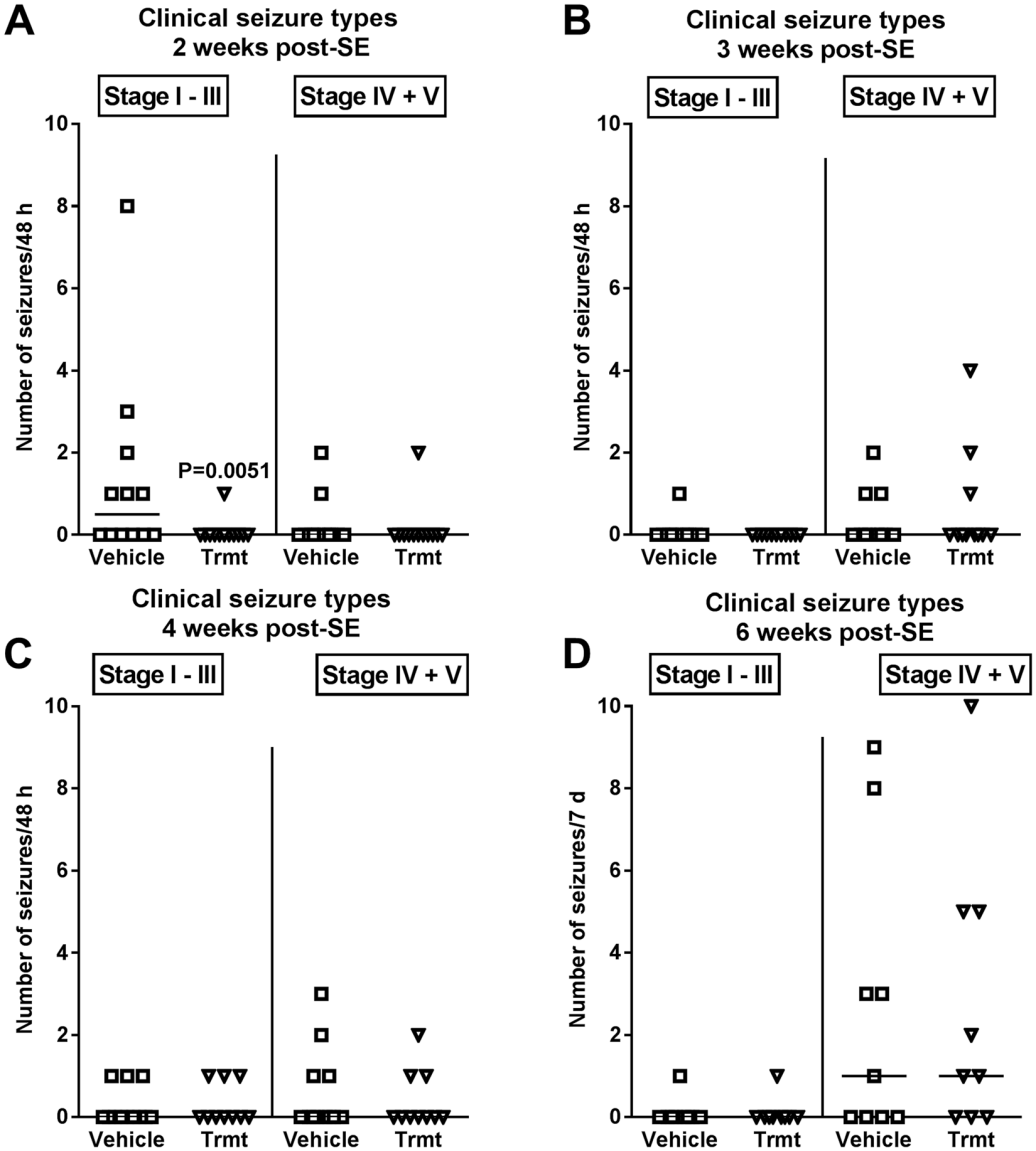
Clinical seizure types occurring at 2, 3, 4, and 6 weeks following intrahippocampal kainate injection in mice and effect of treatment with a combination of NBQX and ifenprodil during the latent period of this model. Data are combined from experiments 2 and 3 (see Fig. 1). In each graph, the number of clinical seizures in individual mice is shown; the group median is indicated by the horizontal line. Seizures are differentiated into focal (stage I-III) and generalized (stage IV and V) seizures. Analysis of data by two-way ANOVA indicated a significant difference between vehicle and drug treated groups (P = 0.0381); *post hoc* analysis by Dunn’s multiple comparisons test indicated that drug treatment significantly reduced seizure frequency at 2 weeks after kainate (P = 0.0051).

However, the effect of treatment with NBQX and ifenprodil on SRS development was only transient, because at 3, 4, and 6 weeks after SE no significant differences to vehicle controls were observed for both clinical (Fig. 2B–D) and electrographic seizures (Fig. 3B–D). We also rated the severity of the clinical seizures by a modified Racine scale (see Methods). Here we found a difference between experiments 2 and 3. In experiment 2, none of the drug-treated mice exhibited generalized convulsive (stage 4 and 5) seizures at 2–4 weeks following SE whereas such seizures were observed in vehicle controls (P = 0.0068), indicating that treatment retarded the progression of seizures (not illustrated). This was not observed in experiment 3. When data of both experiments were combined (Fig. 4), a clear progression from focal (stage 1–3) to generalized convulsive (stage 4–5) seizures was observed in vehicle controls over the 6 weeks following SE. This progression was not significantly retarded by treatment. Two weeks after SE, treatment significantly reduced the frequency of focal seizures (Fig. 4A). In experiment 3, mice were again video-EEG monitored for one week at 12 weeks after SE (Fig. 1); no differences to vehicle controls were observed in seizure frequency or severity (not illustrated).

**Figure 5:**
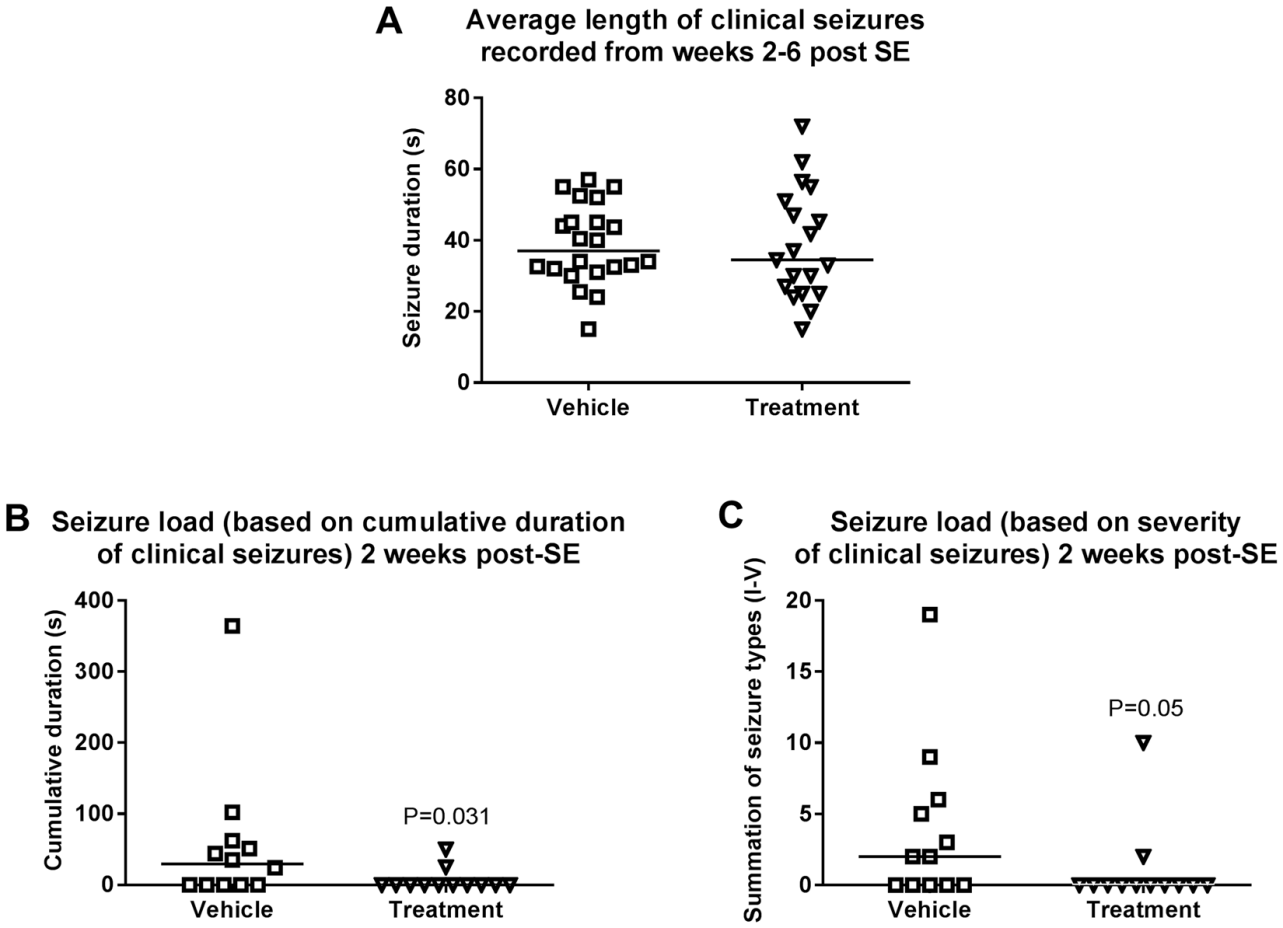
Average length of spontaneous clinical seizures (**A**) and seizure load (**B**, **C**) in the intrahippocampal kainate mouse model and effect of treatment with a combination of NBQX and ifenprodil during the latent period of this model. Data are combined from experiments 2 and 3 (see Fig. 1). In A, the average length of all recorded spontaneous clinical seizures is shown for the vehicle and treatment groups. Because seizure length did not differ at different recording periods (2–6 weeks) after intrahippocampal kainate, data of all recording periods were averaged; the group median is indicated by the horizontal line. In B, seizure load was calculated based on cumulative duration of clinical seizures, wheras in C, seizure load was calculated based on severity of clinical seizures. Only data for 2 weeks after intrahippocampal kainate are shown in B and C, because this was the only recording period where treatment with NBQX and ifenprodil exerted a significant effect (see also Figs 2 and 4).

To further characterize the effect of the drug combination on development of spontaneous seizures after kainate, we determined the duration of the clinical seizures and calculated seizure load both based on duration and severity of seizures. As shown in Fig. 5A, duration of clinical seizures was not affected by treatment. Seizure load was significantly decreased by treatment 2 weeks after kainate, independently of whether seizure load was based on seizure duration (Fig. 5B) or seizure severity (Fig. 5C). At subsequent weeks, seizure load did not differ between vehicle- and drug-treated groups (not illustrated).

**Figure 6:**
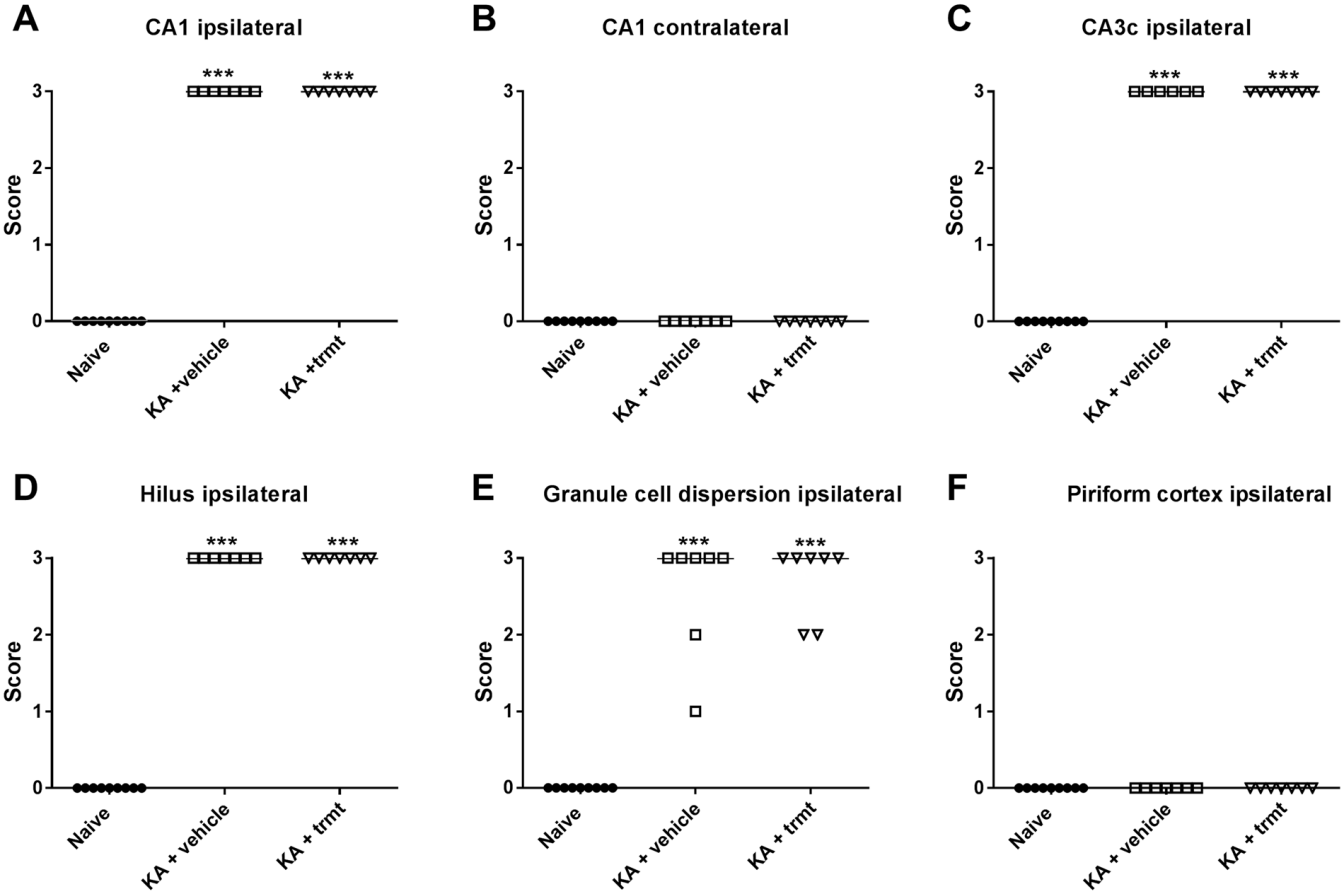
Neurodegeneration following intrahippocampal kainate injection in mice. Data are from mice of groups 2 and 3, which were killed 8–22 weeks after kainate (see Fig. 1). A group of naive mice was used for comparison. Severe neurodegeneration was observed in the ipsilateral hippocampus in CA1 (**A**), CA3 (**C**) and dentate hilus (**D**), but not in the contralateral hippocampus (**B**) and ipsilateral (**F**) or contralateral piriform cortex. In addition to neuronal loss, granule cell dispersion was observed in the ipsilateral dentate gyrus (**E**). Individual data are shown; the group median is indicated by the horizontal line. Significant differences to naive controls are indicated by asterisk (P < 0.001). All data are from a section level of −1.94 mm from bregma (kainate was injected at −2.1).

**Figure 7:**
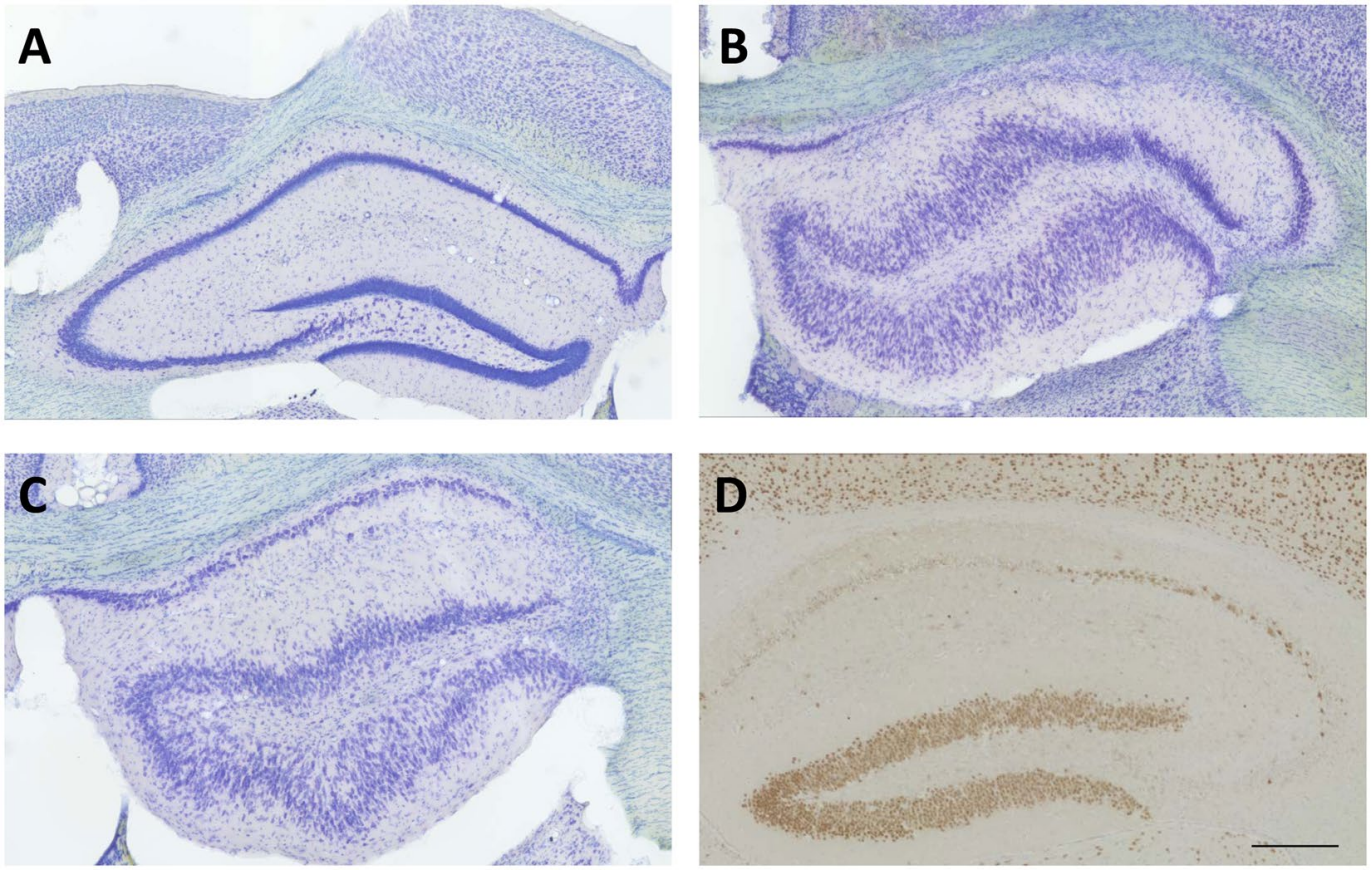
Representative coronal sections (section level −1.94 mm from bregma) of the hippocampal formation of the left (contralateral, **A**) and right (ipsilateral, **B**–**D**) hemisphere of epileptic mice that received unilateral injection of kainate into the right CA1 at −2.1 mm from bregma. A–C are thionin-stained sections from mice of group 3 killed 22 weeks after kainate, while D is a NeuN-stained section of a mouse killed one week after kainate. “A” shows the contralateral (normal) and “B” the ipsilateral hippocampus of a vehicle-treated mouse. “C” shows the ipsilateral hippocampus of a mouse treated with NBQX and ifenprodil. “D” shows the ipsilateral hippocampus of a vehicle-treated mouse. Note the almost complete loss of neurons in CA1, CA3 and dentate hilus in B–D and the marked granule cell dispersion in B and C. One week after kainate, granule cell dispersion was obvious (**D**) but less marked than after 15 weeks (**B** and **C**). Furthermore, ventricles were enlarged in B and C. Scale bar in “D” indicates 200 µm.

At the end of the experiments, mice were killed for brain histology and immunohistochemistry (Fig. 1). As shown in Figs 6 and 7, treatment with NBQX and ifenprodil did not prevent the severe neurodegeneration in the ipsilateral hippocampus induced by intrahippocampal kainate. Almost complete neuronal loss was observed in ipsilateral CA1, CA3c and dentate hilus (Fig. 7B,C). Furthermore, marked GCD was seen, which is characterized by an enlargement of stratum granulosum, a loss of the close apposition between granule cell soma, and the presence of granule cells scattered in the molecular layer with an elongated bipolar change of cell bodies^27^. As shown in Fig. 6E, the severity of GCD in the ipsilateral hippocampus was not affected by treatment with NBQX and ifenprodil. Neuronal damage and GCD were restricted to the ipsilateral hippocampus and extended over an AP area of about −1.22 to −2.80 mm to bregma (kainate was injected at −2.1 mm); no obvious neuronal loss or GCD were observed in the contralateral hippocampus (Figs 6B and 7A) or adjacent parahippocampal regions such as the ipsilateral (Fig. 6F) and contralateral piriform cortex.

The significant retardation of epileptogenesis observed in experiments 2 and 3 prompted us to perform a fourth experiment, in which mice were killed one week after SE (Fig. 1). The aim of this experiment was to evaluate potential mechanisms of the disease-modifying effect of treatment with NBQX and ifenprodil.

### The combination of NBQX and ifenprodil retards granule cell dispersion (experiment 4)

**Figure 8:**
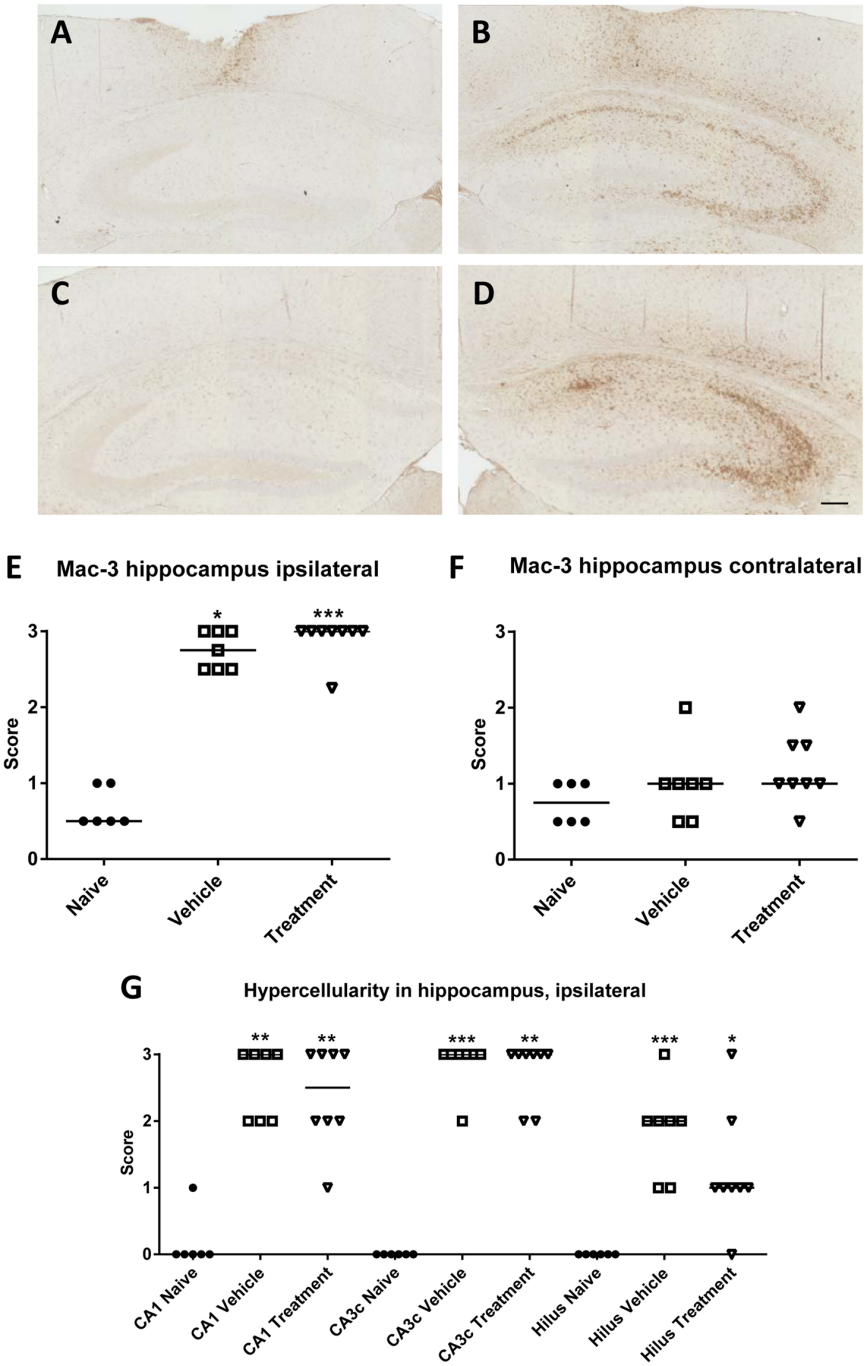
Analysis of Mac-3 positive cells and hypercellularity in the hippocampus at one week following intrahippocampal kainate injection. (**A–D**) Representative photomicrographs showing Mac-3-labeled activated macrophages/microglia at a section level of −1.7 mm from bregma. Serial sections containing the ipsilateral dorsal hippocampus were stained with antibody against Mac-3 in order to label activated macrophages/microglia and scored semi-quantitatively within the hippocampus (see Methods). “A” shows the contralateral and “B” the ipsilateral hippocampus of a vehicle-treated mouse. “C” shows the contralateral and “D” the ipsilateral hippocampus of a NBQX-ifenprodil-treated mouse. Scale bar in “D” indicates 200 µm. Note that Mac-3 staining mainly occurs in the ipsilateral hippocampus, particularly the CA1 and CA3 sectors, and is not affected by treatment with NBQX and ifenprodil. “E” and “F” show semiquantitative analyses of Mac-3-positive cells within the ipsilateral (**E**) and contralateral (**F**) hippocampus. “G” illustrates hypercellularity in different subfields of the ipsilateral hippocampus. In E–F, individual data are shown; the group median is indicated by the horizontal line. Significant differences to naive controls are indicated by asterisk (*P < 0.05; **P < 0.01; ***P < 0.001).

As in experiment 3, no mortality occurred in experiment 4, using the same dosing protocol as in experiment 3 in 8 vehicle controls and 8 drug-treated mice (Fig. 1). Mice were killed one week after kainate, because inflammation is highest at 5–7 days following SE in this model. Our hypothesis was that the significant retardation of epileptogenesis by the NBQX-ifenprodil combination might either be due to an antiinflammatory or neuroprotective effect or both. Activation of microglia and brain infiltration of macrophages, which can be visualized by Mac-3, is a hallmark of the inflammation induced by intrahippocampal kainate^17,49,50^. As shown in Fig. 8B,D,E, marked Mac-3 staining was observed in the ipsilateral hippocampus of vehicle treated mice one week after kainate, but this was not affected by drug treatment. No increased Mac-3-staining was observed in the contralateral hippocampus (Fig. 8A,C,F). In the ipsilateral hippocampus of kainate treated mice, Mac-3 immunopositive cells were particularly observed in those hippocampal areas (CA1, CA3) that were damaged in this model (Fig. 8B,D).

In addition to Mac-3-stained cells, hypercellularity was observed in HE-stained hippocampal sections of kainate-treated mice. Hypercellularity, which can result from both gliosis and inflammatory cells, was observed in ipsilateral CA1, CA3, and dentate hilus and was not affected by drug treatment (Fig. 8G). No hypercellularity was observed in contralateral hippocampal sections.

IL-1β immunostaining showed only few positive cells in the ipsilateral hippocampus of mice one week after kainate (not illustrated). No obvious difference was observed between vehicle treated and NBQX-ifenprodil treated mice.

When degeneration of hippocampal neurons was assessed by immunostaining with NeuN near to the kainate focus (−2.06 to −2.18 mm from bregma) and in more anterior sections of the hippocampus (−1.34 to −1.46 and −1.7 to −1.82 mm from bregma), the neuronal damage determined one week after SE was very similar to that determined several weeks after SE with almost complete loss of neurons in CA1, CA3, and dentate hilus (Fig. 7D). No significant differences in neuronal damage were observed between vehicle-treated and drug-treated mice (not illustrated). Also, when counting neurons in the dentate hilus, no significant inter-group differences in neuron numbers or density were observed (not illustrated).

**Figure 9:**
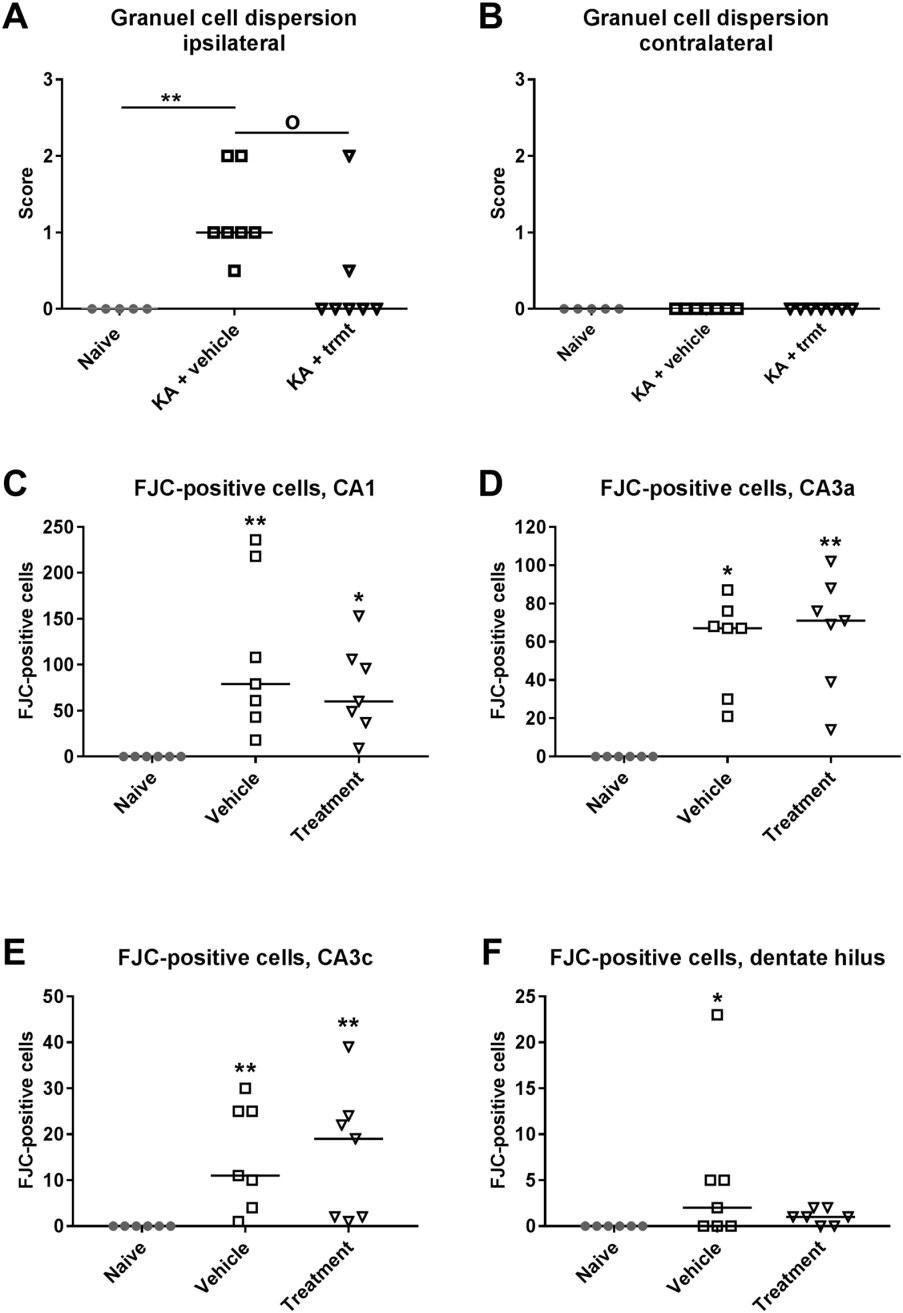
Treatment with a combination of NBQX and ifenprodil during the latent period of the intrahippocampal kainate mouse model reduces granule cell dispersion and number of Fluoro-Jade C (FJC) stained neurons in the ipsilateral hippocampus at one week after kainate. As shown in A and B, granule cell dispersion was only observed in the ipsilateral hippocampus and its severity was reduced by drug treatment. As shown in C–F, FJC-stained neurons were observed in CA1, CA3 and hilus of the ipsilateral hippocampus, but in the dentate hilus only vehicle-treated mice significantly differed from naive controls. In all graphs, individual data are shown; the group median is indicated by the horizontal line. Significant differences to naive controls are indicated by asterisk (*P < 0.05; **P < 0.01), while significant differences between vehicle and drug treated groups are indicated by circle (P < 0.05).

GCD was only moderate one week after kainate (Fig. 7D) compared to the marked GCD seen at 22 weeks after kainate (Fig. 7B,C). As shown in Fig. 9A, treatment with NBQX and ifenprodil significantly reduced GCD compared to vehicle controls. No GCD was observed in the contralateral hippocampus (Fig. 9B).

**Figure 10:**
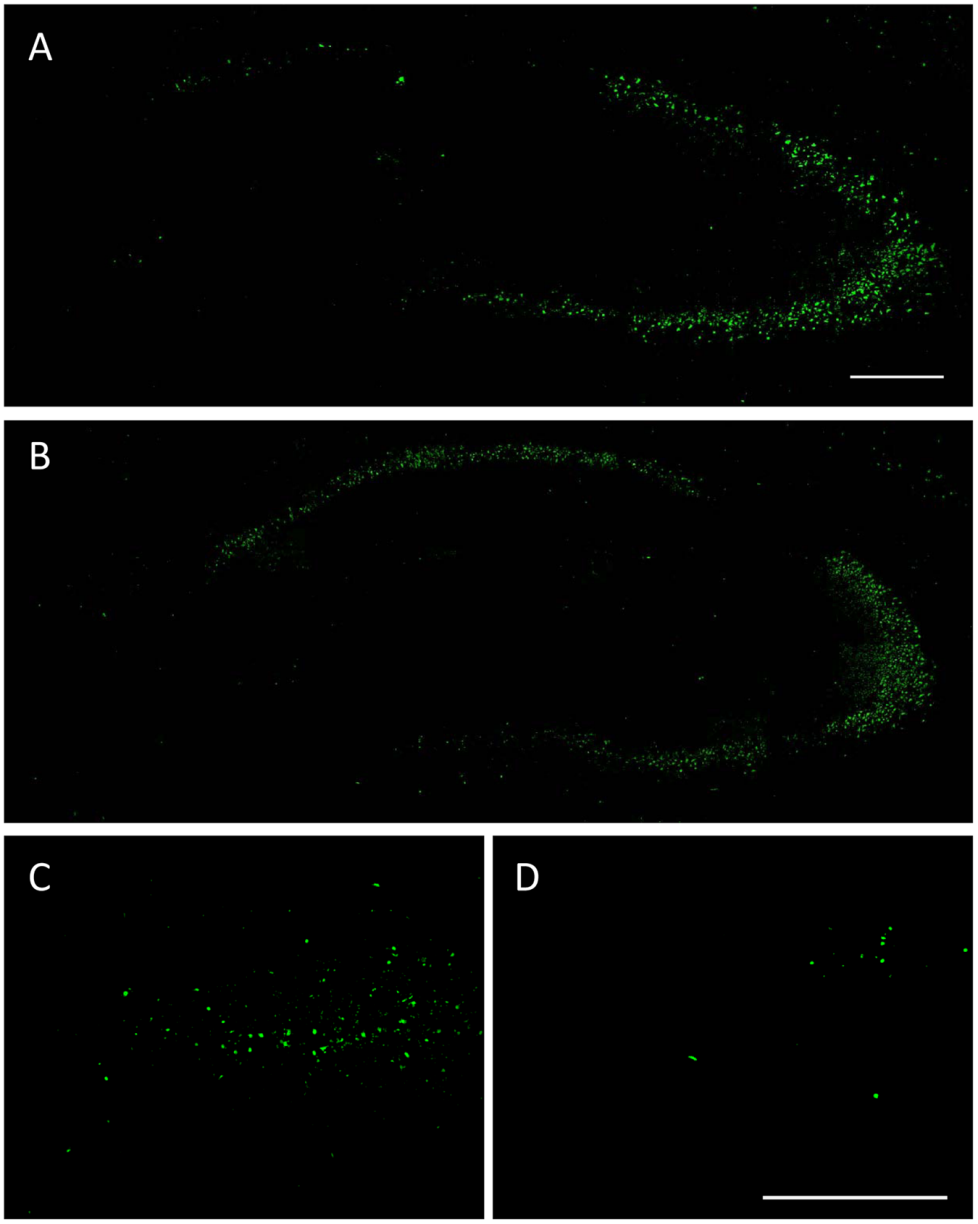
Representative Fluoro-Jade C (FJC) stained coronal sections (section level −1.82 mm from bregma) of the hippocampal formation of the ipsilateral hippocampus of mice that received unilateral injection of kainate into the right CA1 at −2.1 mm from bregma one week before FJC staining. The section shown in “A” is from a vehicle control, while the section shown in “B” is from a drug treated mouse. “C” and “D” are enlarged views of the dentate hilus, illustrating the markedly reduced FJC staining in mice treated with NBQX and ifenprodil (**D**) compared to vehicle control (**C**). The scale bars in “A” and “D” indicate 200 µm.

In addition to assessing histopathological alterations in thionin- or NeuN-stained brain sections, sections were stained with the fluorescent marker FJC, which is extremely specific for degenerating neurons^51^. Staining was not seen in naive controls (not illustrated), whereas in all kainate-injected mice FJC-stained neurons were observed in the ipsilateral CA1, CA3, and hilus (Fig. 10). Most kainate-injected mice showed no or only few FJC-stained neurons in the granule cell layer of the ipsilateral dentate gyrus. In some mice, few stained neurons were also seen in the contralateral hippocampus (not illustrated). Compared to vehicle controls, treatment with NBQX and ifenprodil did not reduce the number of FJC-positive neurons in ipsilateral CA1 and CA3 (Fig. 9C–E). In the hilus, vehicle controls exhibited a significant increase in FJC-stained neurons compared to naive mice, whereas no significant difference to naive mice was observed in mice treated with NBQX and ifenprodil (Fig. 9F; Fig. 10D), which might indicate that the treatment retarded neurodegeneration in this region. However, when vehicle and drug treated groups were compared, no significant difference was obtained between the two groups (P = 0.2257).

## Discussion

Only few previous studies have evaluated rationally chosen drug combinations for antiepileptogenic effects and most of these studies used antiinflammatory or antioxidant drug combinations^52–56^. Prompted by previous studies that showed synergistic anticonvulsant interactions of NMDA and AMPA receptor antagonists in the kindling model of TLE (see Introduction), we evaluated whether such combination provides antiepileptogenic or disease-modifying effects in the intrahippocampal kainate model of TLE. For this purpose, mice were treated with a combination of the AMPA receptor antagonist NBQX and the NMDA antagonist ifenprodil over 5 days, starting 6 h after injection of kainate. The reason for this short treatment period was two-fold. First, the latent period in this model before onset of SRS is only about 5–7 days^29^. Second, for an antiepileptogenic treatment to be clinically manageable, it should be administered when the patient with a potentially epileptogenic brain injury is still hospitalized. Otherwise, compliance may be too low^57^. Thus, the antiepileptogenic effect should be reached after a few days of treatment, or, even better, after one administration. We demonstrated previously that this is not completely unrealistic by showing that one low dose of the NMDA receptor antagonist MK-801 (injected shortly after diazepam) exerts marked disease-modifying effects in the rat kainate model of TLE when injected 90 min after onset of SE^58,59^.

In contrast to traditional NMDA receptor antagonists such as MK-801, ifenprodil acts as an activity-dependent NMDA receptor antagonist that effectively inhibits NMDA receptors activated by high concentrations of glutamate and, at the same time, retains the basal level of glutamate neurotransmission^60^. Ifenprodil is clinically approved for treatment of vascular diseases in several countries because of its alpha-adrenoceptor-blocking properties, while the first AMPA receptor antagonist, perampanel, was approved only recently for treatment of epilepsy^6^. Thus, if a combination of ifenprodil with an AMPA receptor antagonist would exert antiepileptogenic or disease-modifying effects in animal models of acquired epilepsy, this would have immediate translational value, which was a major impetus to perform the present study. Instead of perampanel, we used NBQX as a standard AMPA receptor antagonist, because our previous study on the effects of AMPA receptor antagonism on epileptogenesis was performed with NBQX^8^. In the latter study, treatment with NBQX did not retard or prevent development of spontaneous seizures in the intrahippocampal kainate model and did not reduce the severe neuronal damage occurring in the ipsilateral hippocampus in this model. The NR2B antagonist ifenprodil was chosen for the present experiments because it was shown to counteract the effects of IL-1β and other proinflammatory cytokines^17–20,61,62^, which are thought to play a critical role in epileptogenesis in models of acquired epilepsy, including the intrahippocampal kainate mouse model^20,63^. Furthermore, ifenprodil was reported to exert neuroprotective effects in a rat model of post-SE TLE^22^.

However, finding a tolerable dosing protocol for NBQX and ifenprodil was much more complicated than we expected. Although we tried to avoid toxicity by pretesting the tolerability of the NBQX-ifenprodil combination in epileptic mice, thus using a strategy previously described for other drug combinations^35^, administration of the NBQX-ifenprodil combination during the latent period after intrahippocampal kainate resulted in mortalities, presumably because of respiratory depression that was not observed with even higher doses in the tolerability experiments in epileptic mice. It is well known that SE in rodents is a critical condition, often necessitating specific post-SE care, although this is less a problem in the intrahippocampal kainate model^64^. Dose-dependent respiratory depression is a known adverse effect of AMPA receptor antagonists in rodents^65^, although this was not observed when NBQX alone was administered during the latent period following kainate^8^. An additional problem for tolerability is the fact that ifenprodil is not a selective antagonist of NR2B, but also acts as antagonist of α1-adrenergic receptors, serotonin receptors and calcium channels, suggesting that the therapeutic effectiveness of this compound might be compromised by accompanying cardiovascular interactions^66^. The mortality associated with the administration of the NBQX-ifenprodil combination could not be resolved by changing the type of anesthesia during kainate injection, so that we had to further modify the dosing protocol of the drug combination.

When administered over 5 days, starting 6 h after injection of kainate, the NBQX-ifenprodil combination significantly retarded epileptogenesis in the intrahippocampal kainate mouse model of mesial TLE. To our knowledge, this is the first report of a disease-modifying effect in this model in mice. The disease-modifying effect of the NBQX-ifenprodil combination observed at 2 weeks after kainate was only transient in that all mice exhibited SRS at subsequent video-EEG monitoring periods. Also, the progression of the seizures from focal to generalized was not prevented by the drug combination. Using a similar mouse model, in which SE is induced by unilateral injection of kainate into the amygdala, Iori *et al*.^67^ recently reported that blockade of the IL-1 receptor/Toll-like receptor (IL-1R1/TLR4) pathway by transient administration of a combination of antiinflammatory drugs (VX-765 and the TLR4 antagonist cyanobacterial LPS) *after* epilepsy onset prevented disease progression, but it was not examined whether this drug combination also exerted antiepileptogenic effects when administered during the latent period.

In an attempt to identify the mechanisms of the disease-modifying effect of the NBQX-ifenprodil combination observed in the present study, we examined neuroinflammation and neurodegeneration at one week following intrahippocampal kainate, i.e., after 2 days of washout from the drug treatment. Because activation of microglia and infiltration of peripheral macrophages seem to be key events in this epilepsy model^17,49,50,68^, we used Mac-3 to visualize these cells in the brain. No effect of drug treatment on the increase of Mac-3 stained cells in the ipsilateral hippocampus was observed. Immunostaining of IL-1ß indicated that activation of this cytokine had already disappeared one week after kainate injection. Indeed, studies on the time course of inflammatory changes following intrahippocampal kainate injection in mice have shown that IL-1β and COX-2 are immediately up-regulated in the kainate-injected hippocampus, suggesting that they are early key mediators of neuroinflammation in this model^68^.

Evaluation of hippocampal damage by NeuN-staining did not indicate any neuroprotective effect of the NBQX-ifenprodil combination, although FJC staining indicated some neuroprotective effect in the dentate hilus at one week after kainate. As previously reported^28,36,42^, neuronal degeneration in the ipsilateral hippocampus in response to kainate injection was severe with almost complete neuronal loss in CA1, CA3 and dentate hilus as well as marked GCD, which was not restricted to the immediate area of the kainate injection but extended to more anterior parts of the ipsilateral hippocampus. Treatment with NBQX and ifenprodil did not prevent or reduce GCD determined several months after kainate; however, it retarded the development of GCD as indicated by the significantly reduced GCD observed one week after kainate in drug treated mice. The only other study in which NMDA and AMPA receptor antagonists were administered in the intrahippocampal kainate mouse model examined whether the NMDA antagonist MK-801 (dizocilpine) or the AMPA antagonist GYKI 52466 inhibit the progression of GCD in this model^69^. The latter group found that while treatment with MK-801 after the SE markedly reduced GCD for ≤14 days, GYKI 52466 was not effective in this regard. Neither treatment prevented the loss of principal cells in CA1, CA3 and dentate hilus^69^. Furthermore, as seen in the present experiments with NBQX and ifenprodil, the effect of MK-801 on GCD was only transient^69^. The development of epilepsy with SRS was not examined in the latter study.

GCD in TLE, first described by Houser^70^, is a frequently observed structural abnormality in both TLE patients and TLE models that may play a role in altered dentate gyrus excitability^71^. Unilateral granule cell dispersion is reliably produced in normal mice during the first weeks following unilateral intrahippocampal injections of the glutamate receptor agonist kainate^28^, and it has been shown that expression of the extracellular matrix protein Reelin was dramatically decreased on the kainate-injected side, but not contralaterally^72^. These results suggest that GCD results from decreased Reelin expression, after cell loss or hypermethylation of the Reelin gene^73^. In line with this hypothesis, the expression of Reelin was found to be significantly decreased in tissue samples from TLE patients^74^. Moreover, it was noticed that the extent of GCD dispersion correlated with the extent of decreased Reelin expression. The previous^69^ and present findings that suppression of GCD by glutamate receptor antagonists is not associated with a significant modification of cell loss in CA1, CA3 and dentate hilus suggest that GCD is not closely related to neuronal loss in TLE. Similarly, chronic treatment with rapamycin, an inhibitor of the mammalian target of rapamycin (mTOR) signaling pathway, suppressed GCD in the intrahippocampal kainate mouse model, but did not reduce cell loss or the development of hippocampal electrographic seizures in this model^75^. However, our finding that suppression of GCD coincided with the retardation of SRS development seems to suggest that GCD is involved in epileptogenesis, at least in the mouse model used. Although intrahippocampal kainate injection causes both GCD and epilepsy in mice, perforant pathway stimulation-induced hippocampal injury in mice produces epilepsy without inducing GCD^76^. Furthermore, many TLE patient hippocampi do not exhibit GCD, and other animal models that exhibit confirmed granule cell-onset epilepsy do not show GCD^71^. Thus, the pathophysiological implications of GCD, if any, remain to be clarified.

The present finding that the neurodegeneration was already so severe at 7 days after kainate might indicate that at least part of the cell death was related to immediate neurotoxic effects of the excitotoxic convulsant rather than more delayed and progressive types of cell death observed in models with systemic administration of convulsants or electrical stimulation of limbic brain regions^64,77^. If so, it would not be possible to prevent or markedly reduce the neurodegeneration in the ipsilateral hippocampus by drug treatment several hours *after* injection of kainate. In this respect, models in which kainate is not directly injected into the hippocampus but in the amygdala^78^ or cortical areas adjacent to the hippocampus^79^ and then results in “distant lesions” in the hippocampus (most likely as a result of the kainate-induced SE) might have advantages when evaluating neuroprotective glutamate receptor antagonists such as NBQX or ifenprodil. On the other hand, the present data on FJC-staining of degenerating neurons showed that numerous neurons still degenerated at one week after kainate, indicating delayed processes of cell death. Furthermore, previous experiments with FJC-staining of degenerating neurons in the intrahippocampal kainate mouse model showed that degenerating neurons could be stained in CA1, CA3, hilus and granule cell layer still 26 weeks after kainate injection^42^, possibly as a result of the highly frequent spontaneous electrographic seizures that occur in the ipsilateral hippocampus in this model.

In conclusion, despite several previous reports that NBQX and ifenprodil exert disease-modifying effects in rodent models of TLE when administered alone^9,10,18,19,21,22^, their combined administration did not prevent epilepsy in the intrahippocampal kainate mouse model of TLE, but only retarded epileptogenesis (and GCD) in the majority of the animals. In this respect, it is important to note that our study demonstrates that it is essential to monitor spontaneous seizures at more than one early interval after the epileptogenic insult, because otherwise false positive data on antiepileptogenic drug effects could be obtained. The relatively small impact of treatment with the NBQX-ifenprodil combination on epileptogenesis and the lack of any sustained neuroprotective effect were not in line with our initial hypothesis. Our data do not exclude that this drug combination would result in more promising effects in other models of acquired epilepsy, but a rationally chosen drug combination should have robust efficacy across different models to be a candidate for clinical translation^1^. One may argue that the relatively short duration of treatment (5 days) might have added to the lack of more profound effects on epileptogenesis, but our recent experiments in the same model have shown that the latent period is only about 5–7 days^29^. Furthermore, Lippman-Bell *et al*.^9^ reported that twice daily administration of NBQX (20 mg/kg) over only 2 days following an epileptogenic insult (hypoxia-induced neonatal seizures) significantly attenuated later-life spontaneous seizures in rats. Preliminary data from other rationally chosen drug combinations show that the 5-day treatment protocol used in the present study is capable of inducing long-lasting disease-modifying effects in the intrahippocampal kainate mouse model^80^. Nevertheless, longer treatment with NBQX and ifenprodil may shed further light on the apparent temporal relationship between dentate gyrus reorganization and development of spontaneous seizures. It is important, however, to consider the rapid drug elimination of most drugs in rodents when establishing adequate dosing protocols, because otherwise the drugs may be too shortly maintained in the brain to interfere with the processes of epileptogenesis, thus resulting in false negative data^81^. Lastly, we cannot exclude that the combination of an AMPA receptor antagonist with another NMDA receptor antagonist, such as memantine or ketamine (at subanesthetic doses), acting at other subunits of the NMDA receptor than ifenprodil, would have resulted in a more favorable outcome. In view of the unmet clinical need of epilepsy prevention, it is important to evaluate the pharmacological targeting of mechanisms that are critically involved in epileptogenesis, and glutamate receptor-mediated changes certainly belong to such mechanisms^82^.
